# Learning to Share Health Care Data: A Brief Timeline of Influential Common Data Models and Distributed Health Data Networks in U.S. Health Care Research

**DOI:** 10.5334/egems.279

**Published:** 2019-03-25

**Authors:** John Weeks, Roy Pardee

**Affiliations:** 1 Kaiser Permanente Washington Health Research Institute, US

**Keywords:** HCSRN, OHDSI, VDW, CDM, OMOP, HMORN, i2b2, SHRINE, PCORI, PCORnet, VSD

## Abstract

The last twenty years of health care research has seen a steady stream of common health care data models implemented for multi-organization research. Each model offers a uniform interface on data from the diverse organizations that implement them, enabling the sharing of research tools and data. While the groups designing the models have had various needs and aims, and the data available has changed significantly in this time, there are nevertheless striking similarities between them. This paper traces the evolution of common data models, describing their similarities and points of departure.

We believe the history of this work should be understood and preserved. The work has empowered collaborative research across competing organizations and brought together researchers from clinical practice, universities and research institutes around the planet. Understanding the eco-system of data models designed for collaborative research allows readers to evaluate where we have been, where we are going as a field, and to evaluate the utility of different models to their own work.

## Introduction

A Common Data Model (CDM) is an important part of multi-organization collaborative research. It empowers health care organizations to work together to provide evidence for improving patient outcomes for everyone. The history of well-known CDMs and shared health data networks in health care begins in 1990 with a collaborative project between the National Immunization Program and the Centers for Disease Control and Prevention. The importance of the project was not lost on the organizations involved as they then expanded on that initial endeavor by creating common data models that empowered them to share code and compare analytic results. While the models continue to evolve and many more organizations join the effort to add their curated data to joint research endeavors, the basic subject areas identified for health care research needs remain remarkably consistent.

The models selected here are not a complete list of every shared data resource used in health care research; these models represent the clear majority of shared data resources in the U.S. health care system. The goal is to provide a meaningful picture of the overlapping subject areas that all these resources share.

### Some Terminology

A **Common Data Model** (CDM) is a way of organizing data into a standard structure [1]. A CDM is very useful for health care research because it allows for the pooling of patient information so that comparisons can be made about the relative effectiveness of different treatments.

A **Shared Health Data Network** (SHDN) is a network of organizations that establishes a CDM with data available to the users.

- In a **Pooled SHDN** the data is contributed to a single location where the data is stored and customers access the data in a client/server relationship.
- In a **Federated SHDN** the data is held locally at the originating organizations but implementations are documented in a central clearinghouse, and are available for remote querying. Users at implementing sites can query their own local data or submit their query to the network to get a collective view of the data.
- In a **Hybrid SHDN** data at some nodes is pooled from multiple organizations, while others are single-organization only. From a user’s perspective, these function identically to Federated SHDNs.

A **Distributed Health Data Network** (DHDN) [2] is a shared health data network where data is made available to remote users by way of a query interface. For Federated and Hybrid SHDNs, queries are run at each site and returned either directly to the requester or to a trusted intermediary for compilation and/or comparison.

**Inmon Data Warehouse** was the first broadly accepted data warehouse architecture (Building the Data Warehouse: 1992). It is frequently referred to, by the level of normalization that Bill Inmon recommended be maintained, as “3NF.” 3NF stands for 3^rd^ Normal Form which is a relaxation of the rules that relational databases adhere to. Normal Form is a term introduced by Edgar F. Codd in 1970 when he introduced the relational database model. Over time, the relational model developed stronger and stronger rules that insure data is not replicated in a database. At each level the Normal Form rules insure improved quality data, moving from Normal Form 1 (1NF)—the most relaxed rule—to 6NF—the strictest rule. The cost of applying these rules is that the database is more complex and slower as more rules are placed on the relational data model. The Inmon approach is often called a top down approach to data warehouse design because of the accessibility of its design. Inmon proposed using a relaxed relational model, a pragmatic 3NF data model. This enabled the relational models to run faster under the analytic pressure needed for data warehouses.

A **Kimball Data Warehouse** is a very popular data warehouse model in operational business intelligence organizations. Ralph Kimball introduced dimensional modeling data warehouse design in his book *The Data Warehouse Tool Kit* (1996). It is sometimes referred to as a bottom up approach to data warehouse design because it is focused on the speed and memory usage of the model. The Entity-Attribute-Value (EAV) data model implementation in a relational database often results in a snowflake schema. The star schema is a relaxation of the normalization rules that govern EAV models. This enables a consistent Kimball Dimensional model that makes a more standard interface for generating OLAP cubes. Star schema queries are slow to write and decipher; they are also very efficient from a memory usage and runtime perspective. These features mean that in most situations there are user interfaces that help provide a human readable (tabular) form of the data.

## Sources of Data for the CDM and DHDN

Generally, candidate organizations for implementing CDMs and/or participating in DHDNs are providers of health *care*, health *insurance*, or—in the case of health maintenance organizations (HMOs)—both. These are the organizations that hold data of the types useful for health care services, epidemiology, and health care economics research. In every case, source data comes from multiple systems, including electronic health records (EHRs), Claims (UB 04, CMS1500, etc.), and specialty electronic devices (infusion, radiation, MRI, etc.). The EHRs come from a variety of vendors like Epic, Meditech, and Cerner, or they may be home-grown. One of the primary duties of the individual preparing the data for a CDM is the ability to curate all these data sources and generate a consistent record representing the patient’s medical history. Health care research has very specific expectations of data quality and history availability that is beyond the scope of all business operational requirements. The amount of history available is a key element in funding for many research projects. This curation effort is significant and limits the number of organizations (and their CDMs) who can compete for deep longitudinal research studies.

## A Data Sharing Network for Tracking Treatment Outcomes

### Center for Disease Control (CDC)

#### 2001 – Vaccine Safety Datalink (VSD) Shared Data Network (SDN)

**Figure 1:**
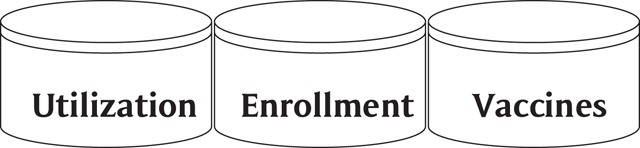
VSD Subject Areas.

In 1990, the CDC launched a collaborative project with several large HMOs to investigate the safety of vaccines [3]. As part of this project the VSD developed a CDM that would be used to share core data [4]. The VSD launched a shared data network in about 2001 with three subject areas (Figure 1): utilization, enrollment, and vaccines.

The VSD SDN is a minimally obfuscated dataset that is pooled at a central location. Each participating organization provides data that helps to create an SHDN where adverse events can be tracked across many areas and diverse populations. The CDC uses its Vaccine Adverse Event Reporting System (VAERS) [5] with this data to report on the relative safety of vaccines.

The VSD uses data files that are roughly organized like an Inmon data warehouse model. They are then sent to the CDC for an analysis of possible adverse events. Hospitals would only use the VSD if they were involved in reporting vaccine events to the CDC. The HCSRN’s VDW model design was influenced by the VSD and that has made it easier for the organizations who have implemented the HCSRN-VDW to generate the VSD data feeds.

## Building Common Data Models to Support Research Across Institutions

### Health Care Systems Research Network (HCSRN)

#### 2003 – CRN publishes the Virtual Data Warehouse (HCSRN-VDW) Common Data Model (CDM)

In 1994, the Health Maintenance Organization Research Network (HMORN) was formed to better translate research findings into care delivery [6]. It consisted of many HMOs with an interest in improving the care their patients received by utilizing and conducting research. From this association came the Cancer Research Network (CRN) in 1999, which was devoted to identifying effective cancer treatments [7].

**Figure 2:**
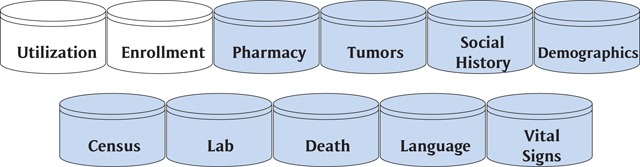
CRN-VDW CDM Subject Areas.

One of the challenges with using a pooled SHDN model like the VSD as the research expanded into cancer research is the amount of personal health information (PHI) that is required. The CRN organizations realized the need for a data source that was held locally but would support sharing code across sites and enable easy aggregation of data from multiple sites to strengthen understanding of research outcomes. The CRN-VDW’s CDM was first published in 2003 and became a standard data model for doing research at the participating HMOs [8]. The current subject areas can be grouped into 11 areas (Figure 2): utilization (patient, provider, procedure, diagnosis), enrollment, pharmacy, tumors, social history, demographics, census, lab, death, language, and vital signs.

In 2015, the HMORN changed its name to the Health Care Systems Research Network (HCSRN). This decision was consistent with the decision to expand the membership to extend beyond HMOs and embrace those who share a commitment to public domain research focused on improving health and health care. The HCSRN states that the VDW is ideally suited for “supporting the development of grant proposals, carrying out preliminary studies, identifying subjects, and assembling study data” [9].

The HCSRN-VDW is closely related to the Inmon data warehouse structure. The Inmon model supports directly readable tabular data, similar to the familiar spreadsheet, which allows analysis without complex query skills. The rules for data curation must be implemented in the extraction, transformation, and loading (ETL) process; the rules can be documented in databases, in external documentation, or in the ETL code itself.

A key benefit of HCSRN membership to VDW implementers is access to a variety of quality assurance scripts that call attention to certain violations of the model (e.g., overlapping enrollment period records for a single person, or use of language codes outside the prescribed set), as well as possible violations (e.g., height measurements outside the plausible range). These scripts are themselves curated by HCSRN along with a process of periodic human review and cross-site comparison, all aimed at ensuring common interpretation/implementation of the CDM, so users can be assured that the data mean the same thing across sites.

## Integrating Shared Data and Analytic and Curation Data Tools

### National Institute of Health (NIH)

#### 2004 – Informatics for Integrating Biology and the Bedside (i2b2)

In 2002, Partners HealthCare System released into production a research data warehouse called the Research Patient Data Registry (RPDR) [10], which consisted of a data model mated with a web-based user interface for querying and inspecting the data. In 2004, the National Institute of Health (NIH) funded grants that were dispersed as National Centers for Biomedical Computing (NCBC) grants. With their grant, Partners HealthCare in Boston, Massachusetts, and Harvard Medical School created an expanded, open-source version of the RPDR application dubbed i2b2 [11].

**Figure 3:**
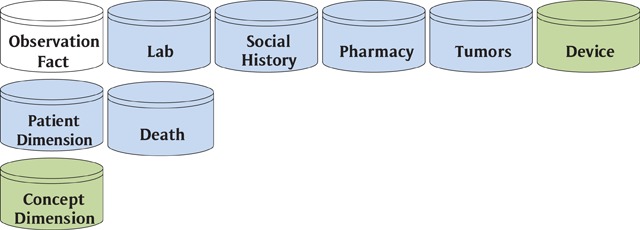
i2b2 CDM Subject Areas.

In addition to effectively providing a CDM in the form of its underlying data model, i2b2’s modular construction and open-source license allowed it to become a full-fledged research data application platform. The i2b2 model is very flexible in accommodating different subject areas in a fact table designed to store observations, in an expanded entity-attribute-value design [12]. A fact table is one of the fundamental structures in the Kimball dimensional model. While its EAV structure allows storage of literally any sort of fact or observation, some of the more frequent subject areas housed in i2b2 instances are (Figure 3) lab, social history, pharmacy, tumors, and device. There is also a patient dimension table that includes death information. Finally, there is a concept dimension that helps to define many attributes in the observation fact table and the management of standard codes.

Unlike the HCSRN’s VDW the i2b2 data is stored in a model akin to the Kimball Dimensional Model which is commonly used in business intelligence reporting. Working with members of the VSD and HMORN VDW, i2b2 provides tools that give greater access to shared data than the VSD. Unlike the VSD, which is dedicated to a specific purpose, i2b2’s SHRINE exploration tool expands the functionality of their data to more general goals such as answering prep to research questions. While i2b2’s primary value proposition was enabling organizations to access their own data, the existence of its CDM fairly begged for a SHDN to be laid over top of it. That capability came by joining their data model and tools to the Shared Health Research Information Network tool (SHRINE) [13]. This tool acts as a cross-organizational interface for i2b2 instances providing members access to the shared data in the network. An advantage that the Kimball model has is that it returns results very quickly. However, the dimensional model queries are not as intuitive to build as the Inmon model. To compensate for this, you will often find that Kimball implementations come with an application front end, like SHRINE for i2b2, that provides a more intuitive way to explore the underlying data.

## An Easy Common Data Model for Clinical Trials

### Observational Health Data Sciences and Informatics (OHDSI)

#### 2007 – Observational Medical Outcomes Partnership (OMOP) CDM

The OMOP CDM was initially published in 2007. The principle of the design was to create a performant data warehouse for studying the effects of medical products [14]. It uses a Kimball Dimensional Model and provides a stack of front end software to make the data more accessible to non-technical users. The original founding partners of the OMOP were the U.S. Food and Drug Administration (FDA), Pharmaceutical Research and Manufacturers of America (PhRMA), and the Foundation for the National Institutes of Health (FNIH) [15]. This group would have been very interested in having a model that would cheaply and easily support the needs of sponsored clinical trials.

**Figure 4:**
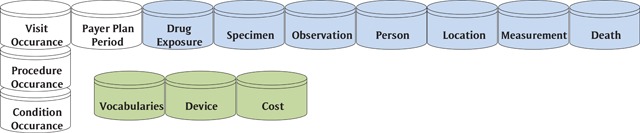
OMOP CDM Subject Areas.

The OMOP CDM shares with the HCSRN-VDW CDM the subject areas: visit/utilization, payer plan period/enrollment, drug exposure/pharmacy, specimen/tumor, person/patient & provider, location/census, measurement (which includes measurements for census & vital signs), death. Additional entities provided with the OHDSI OMOP-CDM are (Figure 4): vocabularies, device and cost. There are other tables generated from the OHDSI core tables, but these are the core subject areas.

Once funding for the OMOP project ran out, OHDSI was formed to continue the work under the Innovation in Medical Evidence and Surveillance program. While OHDSI is the new organizational name, the term “OMOP” lives on in their CDM, the “OMOP Common Data Model” [16].

The OMOP-CDM is built as a Kimball style dimensional model, so the queries run very fast in a relational database environment. They have also compiled tables of standard code values and the relationships between those codes that they call the OMOP Standardized Vocabularies. This creates a powerful data warehouse for exploring health care data with their open source OHDSI technology stack.

## Leveraging Distributed Data Networks to Monitor Drug Safety

### Food and Drug Administration (FDA)

#### 2008 – Sentinel Federated SHDM

**Figure 5:**
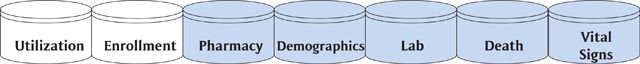
Sentinel CDM Subject Areas.

In 2007, the FDA launched Sentinel to monitor drug safety. Sentinel chose to use the same names for their entities as those used by the HCSRN-VDW. The subject areas in their Distributed Database Common Data Model are (Figure 5) utilization, enrollment, pharmacy, demographics, lab, death and vital signs [17].

The federated SHDN data design is powerful when wanting frequently updated data for monitoring events as they occur [18]. With their data subjects structured very close to the HCSRN-VDW (including the encounter type) it is much easier for networks that want to participate in the Sentinel project if they have an implemented HCSRN-VDW. Sentinel provides a rigorous quality assurance process that can be useful in conjunction with HCSRN-VDW QA scripts for Sentinel participants whose implementations are downstream of an HCSRN-VDW.

## Common Data Models to Connect Research to Health Care Delivery

### Center for Effectiveness and Safety Research (CESR)

#### 2010 – CESR Virtual Data Warehouse (CESR-VDW)

**Figure 6:**
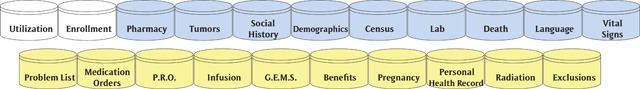
Additional HCSRN VDW Subject Areas.

In 2009, Kaiser Permanente started the Center for Effectiveness and Safety Research (CESR) [19]. As a funding initiative to strengthen and expand the HCSRN VDW at the Kaiser regional research centers (all of which are HCSRN members) CESR began adding new subject areas in 2010 to improve research capabilities and meet the needs of research focused on patient health outcomes. The new subject areas are built to support internal studies as well as external initiatives such as Patient-Centered Outcomes Research Institute (PCORI) and Sentinel [20]. Additional subject areas that have been added to the HCSRN’s VDW include (Figure 6) problem list, medication orders, patient reported outcomes (PROs), infusion, geographically enriched member sociodemographics (GEMS), benefits, pregnancy, personal health record (PHR), radiation, and exclusions.

CESR’s efforts to expand VDW were made with an eye on the other contemporary CDMs that Kaiser regions are participating in—specifically Sentinel and PCORnet. Those latter two CDMs can be derived entirely from CESR’S expanded VDW. This allows any Kaiser region to implement those CDMs by using centrally maintained ETL code. Many of the CESR subject areas are carefully aligned with the tables defined by PCORI’s PCORnet Common Data Model.

### Patient Centered Outcomes Research Institute (PCORI)

#### 2014 – PCORnet Common Data Model

In 2014, PCORI started a common data model/SHDN called PCORnet: The National Patient-Centered Clinical Research Network. Much like Sentinel’s before it, PCORI’s CDM extends the VDW data model with data entities that bear on patient outcomes—particularly patient-reported ones. The PCORnet CDM borrowed from several other data models and works hard to maintain interoperability with standard codes and other CDMs.

**Figure 7:**
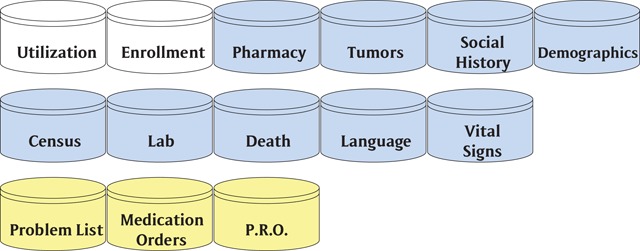
PCORnet CDM Subject Areas.

In addition to providing a publicly available CDM for an expanded set of subject areas (Figure 7), the PCORnet provides Data Quality Checks for their network [21]. In their mission to make patient centered research better and more collaborative, they have created a website that contains many resources for clinical research investigators and data scientists who are interested in learning about their CDM.

## Conclusion

As can be observed in the timelines shown in Appendix A and Appendix B, the many data networks and common models share the same fundamental subject areas. These subject areas are the foundation for health services research and epidemiological surveillance and have withstood decades of use in an era when medicine and data have been changing at a faster rate than at any time in human history. Each of these organizations meet different needs and add to an ecosystem of research tools that increase the ability of data scientists and clinical researchers to work together to identify new and better treatments for patients.

Many of the institutions that have implemented a CDM have built hybrid solutions that incorporate aspects of multiple models that enable them to generate multiple models. The benefits of a hybrid approach is clear when considering the large subject area overlaps between models and the increased opportunity for collaborative research between organizations. By understanding the origins and evolution of the different models, hospitals and research institutes can guide their own choice of model or models that meets their need. Look at the table in Appendix C for a list of the differentiators of each of the models.

More importantly, by understanding how much overlap there is in the data models, the value of an underlying hybrid data model is clear. Once implemented, the organization is free to participate in any kind of research, with the model and cohort that meets the needs of the specific study, by generating the data in the model requested by the funding organization.

## Additional Files

The additional files for this article can be found as follows:

10.5334/egems.279.s1 Appendix A. A Timeline of Common Data Models.

10.5334/egems.279.s2 Appendix B. A Timeline of Shared or Distributed Data Networks.

10.5334/egems.279.s3 Appendix C. A Table of Data Model differentiators.
