# Supplementary figures and images for: Learning to Share Health Care Data: A Brief Timeline of Influential Common Data Models and Distributed Health Data Networks in U.S. Health Care Research
Source: EGEMS (Wash DC). 2019 Mar 25;7(1):4. doi: 10.5334/egems.279 (PMC6437693; doi:10.5334/egems.279)

## Appendix A: A Timeline of Common Data Models

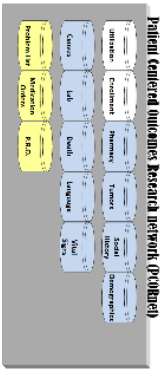

Supplement: Appendix A. — A Timeline of Common Data Models. [file egems-7-1-279-s1.pdf]

Appendix B: A Timeline of Shared or Distributed Data Networks

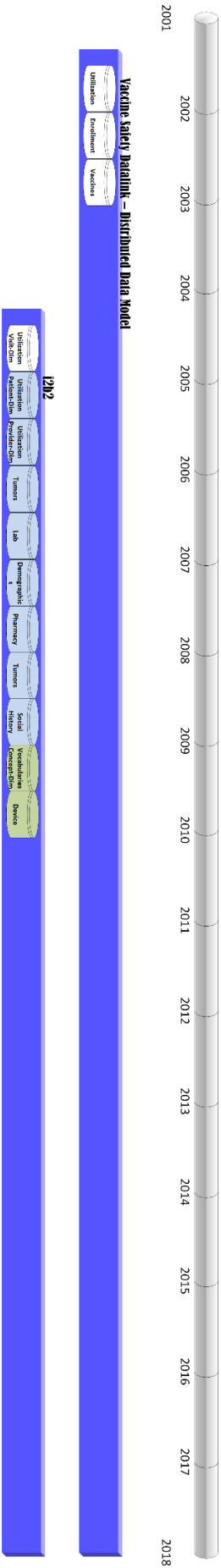

Supplement: Appendix B. — A Timeline of Shared or Distributed Data Networks. [file egems-7-1-279-s2.pdf]
