# Supplementary material for: Learning to Share Health Care Data: A Brief Timeline of Influential Common Data Models and Distributed Health Data Networks in U.S. Health Care Research
Source: EGEMS (Wash DC). 2019 Mar 25;7(1):4. doi: 10.5334/egems.279 (PMC6437693; doi:10.5334/egems.279)
Supplement: Appendix C. — A Table of Data Model differentiators. [file egems-7-1-279-s3.pdf]

Appendix C: A Table of Data Model differentiators

Model	Initial Funder	Current Funder	Warehouse Design Influence	Typical Implementing Organization	Differentiators
**VSD**	CDC	CDC	Inmon: 3NF	HMO	Data pooled at a single site. Used by the CDC to identify adverse events related to vaccines.
**VDW**	NCI	HCSRN	Inmon: 3NF	HMO	Longevity. Implementation guidelines. Mature QA process. Standard code library.
**i2b2**	NIH NCBC	NIH NCBC	Kimball Dimensional	Provider	I2b2 Star Schema is flexible and fast.
**OMOP**	FDA, PhRMA, FNIH	Reagan-Udall Foundation	EAV and Kimball Dimensional	Insurer	Standard vocabulary – EAV style model. Polished (java-based) toolset for user queries. Fast implementation for claims only data.
**Sentinel**	FDA	FDA	Inmon: 3NF	Insurer	Standard querying mechanism & code library. Rigorous QA process.
**CESR**	Kaiser Permanente	Kaiser Permanente	Inmon: 3NF	(Kaiser) HMO	Heavy affinity for Epic EMR-sourced data (as all Kaiser regions have implemented Epic) and other pan-Kaiser data sources.
**PCORnet**	PCORI	PCORI	Inmon: 3NF	Provider	Standard querying mechanism & code library. Rigorous QA process.
